# Integration of neuroscience into psychiatric training and practice: suggestions for implementation

**DOI:** 10.1192/bjb.2024.24

**Published:** 2025-08

**Authors:** Isabel Mark, Norman Poole, Niruj Agrawal

**Affiliations:** 1 South West London and St George's Mental Health NHS Trust, London, UK; 2 St George's University of London, London, UK; 3 South London and Maudsley NHS Foundation Trust, London, UK; 4 Institute of Psychology, Psychiatry and Neuroscience, King's College London, London, UK

**Keywords:** Education and training, neuroscience, clinical neuroscience, biopsychosocial, continuing professional development

## Abstract

Mainstream psychiatric practice requires a solid grounding in neuroscience, an important part of the biopsychosocial model, allowing for holistic person-centred care. There have been repeated calls for better integration of neuroscience into training, although so far with less focus on implementation for life-long learning. We suggest that such training should be accessible and utilised by all psychiatrists, not solely those with a special interest in neuropsychiatry. By considering recent positive developments within the general psychiatry curricula and neuropsychiatric resource implementation, we propose strategies for how this can be progressed, minimising regional disparities within the growing world of virtual learning.

There have been repeated calls for greater integration of neuroscience into psychiatric training and clinical practice. Incremental developments over the past century have improved our understanding of brain mechanisms relevant to a range of psychiatric disorders.^1^ The divergence between neurology and psychiatry, reported by those in the field since the 18th/19th century, with neurologists focusing more on structural neuropathology and psychiatrists shifting to a more psychosocial approach, has led to a gradual reduction in neuroscientific skills among psychiatrists.^2,3^ Within the current framework of the biopsychosocial model in psychiatry and the emphasis on holistic individualised care, this imbalance needs to be readdressed. All psychiatrists would benefit from a stronger core understanding of basic neurosciences, with the equivalent for neurologists to have a psychiatric emphasis in aspects of their training.^4^ In the UK, clinicians working in medical neuroscientific disciplines such as neurology and neurosurgery are already required to have appropriate knowledge and skills in psychiatry to allow better patient care and outcomes, as outlined by the General Medical Council's Generic Professional Capabilities Framework.^5^ Neuroscientific knowledge and skills should likewise be optimally addressed within psychiatry.

Although basic neuroscience makes up a portion of the Royal College of Psychiatrists’ membership (MRCPsych) written examinations, the resulting knowledge and skills are not routinely embedded in continuing professional development (CPD). ‘Cramming’ can be effective for short-term learning, for the purpose of examinations, but not for the long term, resulting in less meaningful application in clinical practice.^6^ Spacing learning over time, interweaving neuroscientific content throughout psychiatric training and CPD, can allow for better educational impact.^7^

For the purpose of this article, the following definitions are used:

- neuroscience: the study of the brain and nervous system and its relevance to mental illness within the biopsychosocial model, aimed at enriching holistic patient care
- neurology: a medical specialty concerned with the diagnosis and treatment of disorders of the nervous system
- neuropsychiatry: a branch of psychiatry focusing on psychiatric aspects of neurological conditions and the disorders at the neurology and psychiatry interface.

## Why is more neuroscience needed within psychiatric training?

Balancing the importance of neuroscience and other biological factors within the biopsychosocial model for best practice has been a controversial topic, subject to much debate. Some argue that psychiatric disorders are primarily disorders of the brain, with ideologies and social practices being woven around core biological concepts, and therefore propose that psychiatrists regain a primarily neurological focus.^4,8^ Others suggest that psychosocial factors should receive more focus in research and practice, arguing that, since the Decade of the Brain in the 1990s, neuroscience has received much investment without delivering significant results or changes in practice.^9^ Most support an approach that develops all aspects of the biopsychosocial model, conceptualising neuroscience in the context of its psychological and environmental interactions.^10,11^ Equal emphasis on all aspects of biopsychosocial care in training is key for developing global understanding and retention of multimodal learning.^11,12^ It allows for individualised person-centred care, a highlight of the new RCPsych curricula, in which a person's needs and values are best met, leading to improvement in patient experience, outcomes, clinicians’ work satisfaction and reduced work-related stress.^13–15^ This concept has recently been expanded by Gómez-Carrillo et al (2023), who suggest that neuroscience should be integrated into psychiatry using a cultural ecosystems approach rather than using pre-existing brain-centric models, promoting sociocontextual and more systemic thinking.^16^

There is of course acknowledgement that most psychiatric disorders have a direct neural component. In these cases, it is crucial for psychiatrists to have sufficient neuroscientific knowledge and skills to facilitate accurate diagnosis, provide meaningful explanations to patients, enable shared decision-making and manage the interface with medical colleagues, leading to better patient outcomes.^1^ The huge surge in research concerning the neuroscientific aspects of psychiatric symptoms and diagnoses in the past few decades has advanced the understanding of depression, psychosis and dementia,^17–19^ the detection and management of autoimmune encephalitis,^20^ as well as having led to emerging pioneering treatments such as deep brain stimulation.^21^ We have observed improved understanding of the interactions between core neurological and psychiatric conditions, such as depression and anxiety in both epilepsy and multiple sclerosis, and psychosis in Parkinson's disease.^22–24^ Neuropsychiatry is often considered the bridging discipline for psychiatry and other brain-based specialties, fostering the integration of neuroscience into patient care, training and research.^2–4,25^ In the future, increased patient needs may overwhelm neuropsychiatry's specialist capacity, where there is already considerable regional variation, resulting in general adult psychiatrists needing to be better equipped to manage these issues (in conjunction with neurology colleagues).^4,26^

The understanding of neuroscience, as well as other medical topics, remains lacking within the profession worldwide, with some psychiatrists and other clinicians previously described as being ‘neurophobic’ and ‘medically deskilled’.^27–29^ Anecdotally we are aware that many psychiatrists in the UK, including trainees, currently feel overwhelmed and lacking in confidence when it is necessary to integrate neuroscientific principles into their clinical practice. With the absence of expectation that neuroscience knowledge and skills should be kept up to date after the MRCPsych has been completed, often combined with the geographical divide between mental and physical healthcare, essential competencies can be lost. The presence of role models and the development of a culture within the profession would be beneficial but can only be developed over time within the construct of RCPsych training and professional development initiatives. Any step forward should of course not minimise the existing emphasis on psychosocial sciences.

In the literature, trainees and training directors have repeatedly called for more neuroscience within psychiatry training, with studies from the UK, USA, Canada and Australia all revealing similar views.^30–33^ The recent survey by Molina-Ruiz et al (2023) of early career psychiatrists from 35 countries again reported a strong preference for more neuroscience training, as well as a better relationship between neurology and psychiatry: 72% acknowledged the extent of regional variation, in part due to political and economic factors, posing the question of how increased training can be practically implemented given current constraints.^26^ Similarly in the UK, in an unpublished survey of 822 psychiatry trainees (54% core trainees, 46% higher trainees) undertaken by the RCPsych Faculty of Neuropsychiatry in 2012, 75.2% expressed an interest in wanting more neuropsychiatry training even though only 25% wished to pursue neuropsychiatry as a career choice, highlighting the acknowledged relevance of neuroscientific skills for all psychiatrists regardless of intended subspecialty.

## How might psychiatry training be modified to integrate more neuroscience?

Proposals for better integration of neuroscience into psychiatry training date back 50 years, with previous calls for neuropsychiatrists to lead on this issue.^1,4,10,30–32,34,35^ Box 1 illustrates some of the global initiatives to date, mostly aimed at improving resources for those wishing to improve their neuroscientific knowledge and skills in their own time and having an existing specialist interest in the subject. Although these measures have been successful to an extent, this article argues that without fully embedding neuroscience within the curriculum, they may not engage and be inclusive for all learners regardless of subspecialty, particularly those not naturally neurologically inclined.

Box 1 Global initiatives focused on integrating neuroscience into psychiatry training

- The Psychiatry Milestone Working Group in the USA developed suggested clinical milestones for general psychiatry training, although concerns have been expressed about how such a competency framework would be implemented given regional variation.^36,37^
- The National Neuroscience Curriculum Initiative (NNCI) in the USA, established in 2013, provides open-access resources for teaching to make neuroscience more relatable and accessible to psychiatry trainees.^38^
- The Gatsby Project in the UK, established in 2016,^39^ is a Wellcome Trust project aimed at developing and embedding neuroscience within the Royal College of Psychiatrists’ membership examination (MRCPsych) curriculum, including the following initiatives:
  - developing additional continuing professional development (CPD) online and TrOn (Training Online) modules with neuroscience content
  - the introduction of Brain Camps, 1-day events for educators with a refresher on brain-based research and workshops on relevant teaching strategies
  - an annual RCPsych neuroscience conference.
- There are many independent collaborations and publications directing readers to resources,^40,41^ with suggestions including:
  - websites such as BrainFacts.org, sharing accurate and up-to-date knowledge in neuroscience with the public
  - YouTube series such as Neuroscience Crash Course (by the British Neuroscience Association) and 2-Minute Neuroscience (by Neuroscientifically Challenged)
  - podcasts such as the MQ Open Mind (by MQ: Transforming Mental Health)
  - webinars such as BRAINCAST (by Maudsley Learning), which offers weekly sessions on key topics with interviews with leading experts in the field
  - TED talks: a large variety on understanding the brain.

Efforts thus far have been limited by several barriers, noted both in the UK and internationally. Particular obstacles have been a paucity of leading experts who might teach and impart knowledge, regional disparities challenging reliable clinical exposure, limited specified requirements within the psychiatry curriculum and a lack of drive among some general psychiatrists to continue to develop and embed neuroscientific skills into practice.^25,38,42,43^

Developments in online learning potentially offer a solution. A ‘blended’ learning regime (a combination of face-to-face opportunities with online teaching and resources made necessary by the COVID-19 pandemic) has become the accepted norm and complements the emergence of hybrid working.^44^ There are fewer time constraints on teachers and learners and a variety of andragogic methods available with guidance for best practice.^44,45^ More centralised learning is now possible, with the option of creating regional hubs to direct learning opportunities and offer appraisal. Learners who do not have neuropsychiatrists in their training location can now access hybrid teaching sessions, clinical exposure opportunities, remote supervision and an array of online learning resources.^43^ Work undertaken by the National Neuroscience Curriculum Initiative (NNCI) and Gatsby Project (Box 1), as well as other independent groups, can be utilised, further developed and used to inspire additional initiatives.^38,39^ Developing a culture of engagement and interactivity is key, with senior psychiatrists and educators reinforcing these links. Advocacy within overarching psychiatric and medical organisations is a crucial component to prioritise neuroscience education and set expectations for the level of knowledge and skills required, with endorsement and support of the new learning opportunities available.^43^

## Suggested template for the reintegration of neuroscientific principles into psychiatric training, continued learning and clinical practice

Considering international successes, barriers and developments to date, a template is suggested below for how neuroscience can be best integrated into life-long learning for psychiatrists. Although the UK is used as a case example, integration principles and strategies can be similarly applied internationally.

### Consideration of learning needs and competencies

As outlined above, the rapidly evolving advances in the neuroscientific understanding of psychiatric disorders in parallel with the lack of adequate opportunities to acquire neuroscientific skills highlights the need to restructure and rebalance psychiatry training. A detailed consensus regarding what basic neuroscientific and clinical neuropsychiatric competencies are required has been developed through the Psychiatry Milestone Working Group, with similar recommendations from the Gatsby Project, as outlined in Box 1.^36,39^ Both are reflected in the new RCPsych curricula to an extent, although some recommendations lack specificity and clear guidance regarding implementation.^15^ Box 2 highlights the key recommended learning outcomes concerning neuroscience-related capabilities within the new RCPsych curricula.

Box 2 Key neuroscience-related capabilities^46^

- ‘During core training it is recommended that trainees gain experience of two psychotherapeutic modalities as well as experience working with patients across the lifespan […] and with those with neurodevelopmental conditions'
- ‘Consistently demonstrate effective communication approaches with patients and relevant others, including those with neurodevelopmental disorders [...]'
- ‘Demonstrate an appropriate understanding of a person-centred holistic approach to mental disorders, including a knowledge of developmental [ … ] genetic and epigenetic risks (including resilience and vulnerability factors) and neuro-biological influences on mental disorders)’
- ‘Receive a full psychiatric history from, perform a Mental State Examination (MSE) on and assess capacity of patients within a range of mental and neurodevelopmental disorders across the lifespan, in routine, urgent and emergency situations incorporating appropriate terminology'
- ‘Conduct a thorough physical examination [including neurological], undertaking relevant physical investigations and take responsibility for acting on your findings in a timely fashion’

### Consideration of learning opportunities at all stages of training

All levels of training must be considered to better integrate neuroscientific knowledge and skills into psychiatric practice. Suggestions for integrating neuroscience training at various career points are listed in Table 1.

**Table 1:** Options along the UK psychiatry training pathway

Suggested interventions for consideration	Career stage	Location of delivery	Method of delivery
Combined neurology and psychiatry sessions as part of existing teaching programmes	U, FY, CT	Local	Face to face, eLearning
A shared foundation neurology/psychiatry placement	FY	Regional	Face to face
Popularising FY taster weeks within neuropsychiatry teams	FY	Regional	Face to face
Neuroscience foundation course for those interested	FY, CT	Local, regional	Face to face, eLearning
Local MRCPsych courses to contain more neurology cases, with clear examples on how principles can be applied to routine practice	CT	Local	Face to face, eLearning
Regional/national neuroscience sessions, in conjunction with RCPsych neuropsychiatry faculty members, offered within local MRCPsych courses, either ‘live’ or recorded for later asynchronous study	CT, ST	Regional, national	eLearning
eLearning options suggested within local programmes, already developed by several, including the Gatsby project	CT, ST	National	eLearning
Neuro case discussion groups (similar to Balint groups), with potential for integration with neurology trainees	CT, ST	Regional	eLearning
Neuropsychiatry placements (or fellowships) to be optimised	CT, ST	Regional	Face to face
Special interest days within neuropsychiatry or neurology teams, to include options for attending virtual patient workshops	ST	Regional	Face to face, eLearning
Trust academic programmes to include specific recommended neuroscience sessions, again potentially in conjunction with RCPsych neuropsychiatry faculty members	CT, ST, CPD	Local	Face to face, eLearning
Dean's Grand Rounds at the RCPsych	CPD	National	eLearning

MRCPsych, Royal College of Psychiatrists’ membership examination; U, undergraduate; FY, foundation years; CT, core psychiatry training; ST, higher/specialist psychiatry training; CPD, continuing professional development for those not in training.

Combining teaching for brain-based specialties as early as medical school might affect how all clinicians view mental health within medicine as a whole, with previously reported success in combining neurology and psychiatry placements.^47,48^ As well as joint placement options, integrating teaching across Foundation Years is another option for establishing collaboration across disciplines.

Local psychiatry teaching programmes (often referred to as MRCPsych courses) provide a key opportunity for scaffolding the knowledge and skills of core trainees, with neuroscientific teaching potentially involving expert-delivered sessions online across regions, thereby improving consistency and removing geographical disparities.^49^ Additional eLearning and external courses or conferences could be highlighted in MRCPsych courses for those who wish to develop skills further, endorsed by local training directors. Offering ‘neuro case discussion groups’ to core and specialist psychiatry trainees, where case formulations with a neurological (or biological) component are developed, would be possible. Appropriate supervision can be offered virtually where there is a paucity of experts.^34,37^ Such groups might highlight how best to integrate all perspectives (from biological to psychosocial), validating each other to advance the holistic and global perspective of the clinical case discussed.^50^ Close collaboration between psychiatrists, other clinicians in the field of neuroscience and non-clinical researchers might further support this process, as well as offering mentorship and promoting further academic opportunities.^51^ Neuropsychiatric, neurological or medical placements could also be optimised at all trainee levels, with additional options of special interest or taster sessions, thereby creating opportunities for wider clinical exposure with local trainer endorsement.^34,52^ Joining neuropsychiatry patient assessments or group sessions remotely might be another option, with appropriate consent and organisational support.

Learning opportunities need not be limited to training levels but should support CPD for all clinicians. Regular neuroscientific sessions within a trust's academic programmes can be optimised, with local programmes supporting trainees to attend other regional programmes (e.g. at regional neuroscience centres) remotely if these sessions are unavailable. Additional online national options can keep neuroscientific knowledge and skills up to date, as well as applying these to clinical practice, as seen in recent initiatives such as the introduction of Dean's Grand Rounds and the Enhancing Generalist Skills programme (‘enhance’).^53,54^ Formalised credentialling for neuropsychiatry may open the route for an accredited path to acquiring neuropsychiatry and neuroscience experience in the future.

Any proposed changes, of course, need to fit into the existing RCPsych curricula,^46^ which state that all neuroscience training or exposure within psychiatry should be considered with an equal emphasis on psychosocial aspects of care, all part of a holistic psychosocial model. As the current focus is tailoring education to individual need, the option of introducing additional mandatory neuroscience requirements does not appear viable. The focus therefore needs to be on optimising opportunities, making them easier to access, of sound quality and thereby more appealing. An eventual change in attitude, expectation and culture is the overall aim, with trainees wanting to embed the knowledge and skills learned for exams into their life-long practice. We believe that this can be achieved in time, with senior psychiatrists leading by example, ideally with RCPsych endorsement, routinely considering neuroscientific principles within their clinical practice and demonstrating how others might do the same.

### How assessment might be reviewed to ensure sustained learning

The current MRCPsych examination, used in the UK and often internationally, already has a considerable neuroscience component within Paper A and we acknowledge the success of this. However, to ensure longer-term impact and effective scaffolding of concepts, further assessment may need to be introduced as training progresses. Workplace-based assessments (WPBAs) offer the most feasible way of doing this, as they are already an integral part of the psychiatry curriculum and there is a requirement that these must be completed for end-of-year appraisals. The new placement-specific personal development plans (PDPs) offer a tailored approach to help trainees acquire clinical experience and expertise in areas required by the curriculum.

We offer four main suggestions of how this may be considered within psychiatry training and ongoing career development. First, there could be a requirement or recommendation that trainees complete one clinical WPBA per year that has a biological or neuroscientific focus, as part of a holistic formulation, with monitoring and review of this at the annual review of competence progression (ARCP). Monitoring progress within clinical professional development could be similarly addressed during appraisal, such as within a case-based discussion (CbD), with a requirement for one case in each revalidation cycle to have a neuroscientific perspective. Second, as core trainees are already entitled to a case-based discussion group assessment (CBDGA) focused on psychotherapy during WPBA, an additional CBDGA could be introduced involving a case that the core trainee presents with a medical or neuroscientific perspective and formulation, having already discussed it in one of the new neuro case discussion groups outlined in Table 1. Third, new stations could be introduced as part of the clinical assessment of skills and capabilities (the CASC), the final component of the MRCPsych examinations, including some to specifically develop formulation skills with a neurological component, or explain a neuroscientific investigation, diagnosis or management plan to a patient or carer. Finally, a reflective write up of a physical health (ideally neuroscientific) problem within a psychiatric case could be introduced as a requirement for completion of general psychiatry training, offering an appropriate and balanced equivalent to the psychotherapy short and long cases that are already present in the curriculum. As with previously suggested initiatives, developments in online working may make identifying appropriate assessors (whether neuropsychiatrists, general psychiatrists with a neurology interest or neurologists with a mental health interest) more achievable.

## Conclusion

This article discusses how best to reintegrate neuroscientific principles into psychiatric training and life-long learning, an issue that has recently been acknowledged to be an important area for development for early career psychiatrists.^26,30^ Despite several notable successes to date, this has been a considerable challenge, given the vast scope of the topic and the regional variability of expert resource provision. Development of online teaching and working can allow educators to standardise training and opportunities. We must also now shift our focus to implementing learning opportunities for all psychiatrists regardless of subspecialty, not solely those with a special interest in neuropsychiatry. A larger focus on neuroscience within training should help provide the future psychiatry workforce with the necessary tools to be able to consider the psychiatric patient from a brain-based (biological) point of view, thus giving appropriate weight to the biological part of the biopsychosocial model that has been missing for some psychiatrists in recent decades, thereby optimising person-centred care.

Future plans will require a wider conversation, with collaboration and further discussion among all relevant professionals and educational leads. Joint working with various colleagues working in neuroscientific disciplines, including neurology, would be desirable. An upskilled psychiatric workforce could lead to improved recruitment rates, job retention and job satisfaction as well as, importantly, a standardisation of patient care on a national and even international level.

## Data Availability

Data availability is not applicable to this article as no new data were created or analysed in this study.

## About the authors

**Isabel Mark** is a consultant psychiatrist in general adult psychiatry at South West London and St George's Mental Health NHS Trust, London, UK and an honorary senior lecturer at St George's University of London, London, UK. **Norman Poole** is a consultant neuropsychiatrist at the Lishman Unit, Bethlem Royal Hospital, South London and Maudsley NHS Trust, London, UK and a senior clinical lecturer at King's College London, London, UK. **Niruj Agrawal** is a consultant neuropsychiatrist at South West London and St George's Mental Health NHS Trust, London, UK and an honorary senior lecturer at St George's University of London, London, UK.
